# Clonal Groupings in Serogroup X *Neisseria meningitidis*

**DOI:** 10.3201/eid0805.010227

**Published:** 2002-05

**Authors:** Sébastien Gagneux, Thierry Wirth, Abraham Hodgson, Ingrid Ehrhard, Giovanna Morelli, Paula Kriz, Blaise Genton, Tom Smith, Fred Binka, Gerd Pluschke, Mark Achtman

**Affiliations:** * Swiss Tropical Institute, Basel, Switzerland; † Navrongo Health Research Centre, Ministry of Health, Navrongo, Ghana; ‡ Max-Planck-Institut für Infektionsbiologie, Berlin, Germany; § University of Heidelberg, Heidelberg, Germany; ¶ National Institute of Public Health, Prague, Czech Republic

**Keywords:** molecular epidemiology, genotyping, meningococcal meningitis, Africa

## Abstract

The genetic diversity of 134 serogroup X *Neisseria meningitis* isolates from Africa, Europe, and North America was analyzed by multilocus sequence typing and pulsed-field gel electrophoresis. Although most European and American isolates were highly diverse, one clonal grouping was identified in sporadic disease and carrier strains isolated over the last 2 decades in the United Kingdom, the Netherlands, Germany, and the United States. In contrast to the diversity in the European and American isolates, most carrier and disease isolates recovered during the last 30 years in countries in the African meningitis belt belonged to a second clonal grouping. During the last decade, these bacteria have caused meningitis outbreaks in Niger and Ghana. These results support the development of a comprehensive conjugate vaccine that would include serogroup X polysaccharide.

Bacterial meningitis due to *Neisseria meningitidis* (meningococcus) causes epidemics in Africa usually associated with serogroup A meningococci. Sporadic cases, outbreaks, and hyperendemic disease in Europe and the United States are usually caused by serogroups B and C (*1*). Occasionally, however, endemic disease and outbreaks are caused by bacteria belonging to other serogroups, including W135, Y, and X. Serogroup X *N. meningitidis* was described in the 1960s (*2**,**3*), and serogroup X meningitis has been observed in North America (*4*), Europe (*5**,**6*), Australia (*7*), and Africa (*8**,**9*). Serogroup X outbreaks have been reported in Niger (*10**,**11*) and Ghana *_(12)_*. In some cases, serogroup X disease has been associated with a deficiency of particular complement components (*13**,**14*) or with AIDS (*15*).

Asymptomatic nasopharyngeal carriage of *N. meningitidis* is common, and in only a small percentage of colonized persons do the bacteria invade the bloodstream and cerebrospinal fluid to cause disease. Meningococcal populations are highly diverse, and lineages of meningococci with increased capacity to cause invasive disease are thought to arise periodically and spread, sometimes globally (*16*). Relatively few of these hyperinvasive lineages or clonal groupings are responsible for most meningococcal disease worldwide (*17*). These clonal groupings diversify during spread (*18*,*19*), primarily as a result of frequent horizontal genetic exchange (*19*–*21*). However, many variants are isolated only rarely or from a single country and are not transmitted further because of bottlenecks associated with geographic spread and competition (*19*,*22*). The population structure of *N. meningitidis* is effectively panmictic as a result of frequent horizontal genetic exchange (*23*), but that of some groupings, such as epidemic serogroup A meningococci, is largely clonal (*24*). The population structure of serogroup X meningococci has not yet been investigated in detail.

After an epidemic of serogroup A disease in 1997-1998 in northern Ghana (*9*), we conducted a longitudinal carriage study to investigate the dynamics of meningococcal carriage during an interepidemic period (*12*). We observed a sharp increase in nasopharyngeal carriage of serogroup X meningococci by healthy persons, accompanied by several cases of serogroup X meningitis. To investigate the phylogenetic relationships of these bacteria, we compared the isolates from Ghana with other serogroup X meningococci isolated during recent decades in Africa, Europe, and North America.

## Materials and Methods

### Bacterial Strains

We analyzed 134 *N. meningitidis* isolates of serogroup X by pulsed-field gel electrophoresis (PFGE) (130 isolates) or multilocus sequence typing (MLST) (41 isolates). Of these bacteria, 102 were isolated in Africa from 1970 to 2000: from meningitis patients (9 isolates) and healthy carriers (70 isolates) in Ghana, 1998-2000; from healthy carriers in Mali in 1970 (9 isolates) and 1990-91 (4 isolates); and from patients in Chad (1995, 1 isolate), Niger (1997-1998, 4 isolates), and Burkina Faso (1996-1998, 5 isolates). Six isolates were not tested serologically; the other 96 were NT:P1.5.

In addition, 32 serogroup X strains isolated from 1988 to 2000 in the United Kingdom (22 isolates), Germany (3 isolates), United States (4 isolates), France (1 isolate), Norway (1 isolate), and the Netherlands (1 isolate) were included in the analysis. The 26 strains subjected to further testing had diverse serotypes (2b, 4, 4/21, 14, 16, 21, 22) and serosubtypes (nonsubtypable [NST]**,** P1.5, P1.5,10, P1.7, P1.12, P1.14, P1.15, P1.16) in various combinations.

### Molecular Typing of Bacteria

PFGE was done by digesting chromosomal DNA prepared in agarose blocks with *NheI* and *SpeI* as described (*22*), and MLST by sequencing gene fragments of *abc*Z, *adk*, *aro*E, *fumC*, *gdh*, *pdh*C, and *pgm*, also as described (16; http://www/mlst.net). The detailed MLST results and sources of isolates have been deposited in a public database http://www.mlst.net). Additional MLST data for 31 isolates in 30 sequence types were obtained with permission from http://www.mlst.net.

### Data Analysis

A neighbor-joining tree was constructed by using the numbers of MLST allele differences with Bionumerics 2.0 *(25)*.

## Results

**Figure 1:**
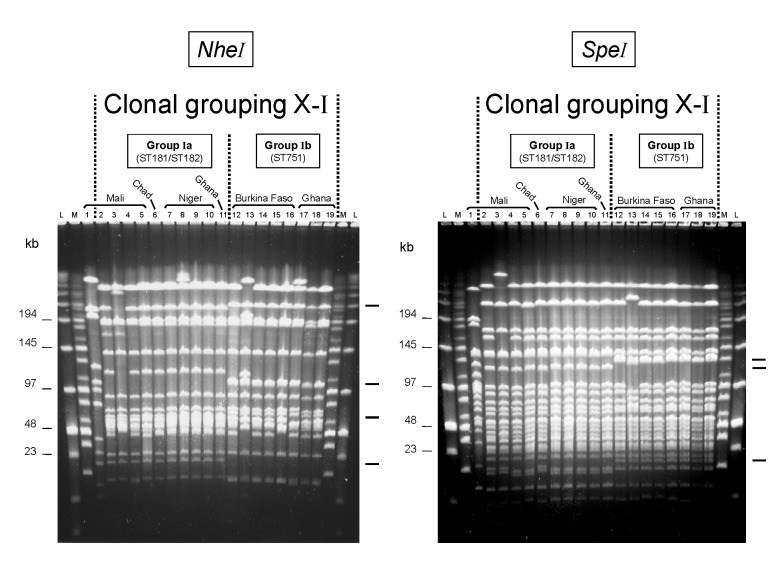
Two groups of pulsed-field gel electrophoresis patterns among *NheI-* and *SpeI-*digested chromosomal DNA from selected serogroup X *Neisseria meningitidis* strains isolated in Africa. Lane: strain: 1: D93 (ST188); 2: 1970; 3: 3187; 4: 3529; 5: D5; 6: LNP13407; 7: LNP14964; 8: LNP15040; 9: 97013; 10: 97014; 11: Z9413; 12: LNP14297; 13: LNP15061; 14: BF2/97; 15: BF5/97; 16: BF1/98; 17: Z7091; 18: Z8336; 19: Z9291. Molecular weight markers were loaded in the flanking lanes as indicated (L: low-range marker; M: midrange marker); their molecular weights are indicated at the left. Characteristic band differences are indicated on the right.

**Figure 2:**
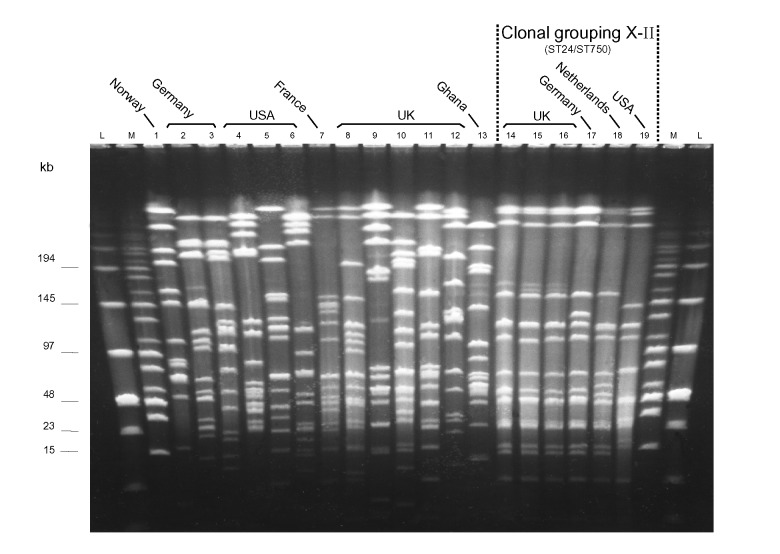
Pulsed-field gel electrophoresis patterns of *NheI*-digested chromosomal DNA of selected serogroup X *Neisseria meningitidis* isolates from Europe and the United States, plus a prototype isolate from Ghana (lane 13). Lane: strain: 1: E26; 2: X4571; 3: X4890; 4: M2526; 5: M4222; 6: M3772; 7: LNP17351; 8: J88-603; 9: K89-1395; 10: L92-1413; 11: M98-253172; 12: M00-240465; 13: Z9291; 14: M98-252848; 15: M98-252718; 16: M99-240899; 17: X5967; 18: 860060; 19: M4370.

PFGE with two discriminatory rare-cutting enzymes *NheI* and *SpeI*) was used to identify groups of closely related strains in 134 isolates of serogroup X *N. meningitidis* from countries in Africa, Europe, and North America. All but 3 of 102 isolates from Africa had similar PFGE patterns (Figure 1, clonal grouping X-I). In contrast, 19 of 32 isolates from Europe and North America had distinct PFGE patterns (Figure 2) that differed from those of the African isolates. However, among the latter 32 strains, similar PFGE patterns were observed for 13 isolates from the United Kingdom, Germany, the Netherlands, and the United States (Figure 2, clonal grouping X-II).

**Figure 3:**
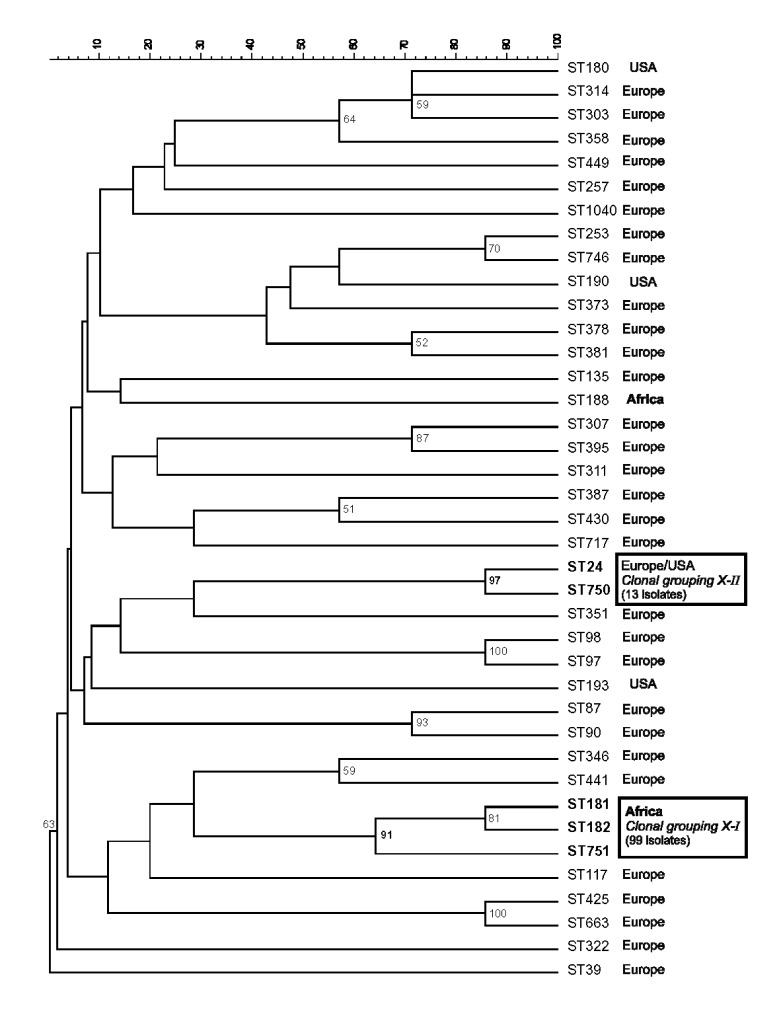
Neighbor-joining phenogram of allelic identities in 39 multilocus sequence typing ^(^MLST) sequence types from serogroup X *Neisseria meningitidis*. Numbers at nodes are the percentages of 1,000 bootstrap replicates in which these nodes appeared. Only nodes with percentages >50% were included. The two boxes indicate clonal groupings that were detected by MLST or pulsed-field gel electrophoresis.

Forty-one isolates, each representing a distinct PFGE pattern, were analyzed by MLST. For bacteria from which multiple isolates with a similar PFGE pattern had been detected, we tested at least one representative from each year and country of isolation. Together with other data in the MLST WEB site (http://www.mlst.net), 39 distinct sequence types (STs) have been found in 50 serogroup X meningococci. The general structure of a neighbor-joining tree of allelic differences resembles a bush, with little phylogenetic structure (Figure 3). However, isolates with similar PFGE patterns were assigned to closely related STs. All 29 clonal grouping X-I isolates analyzed by MLST were in STs ST181, ST182, or ST751 (Figure 3), which differ by one to three of the seven gene fragments (Table). Similarly, all five clonal grouping X-II isolates were in STs 24 and 750, which differ by one of the seven gene fragments (Table). The three unusual African isolates (strain designations D87, D91, and D93) were in ST188, which is very distinct from STs of clonal grouping X-I (Figure 3). These results show that numerous serogroup X isolates from Africa and nearly half the serogroup X isolates from Europe and North America belong to two clonal groupings, while other serogroup X isolates from Europe or North America are quite diverse.

**Table:** Multilocus sequence typing results of two serogroup X *Neisseria meningitidis* clonal groupings

ST	Allele numbers							Country (no. of isolates)	Year
	*AbcZ*	*Adk*	*aroE*	*FumC*	*gdh*	*pdhC*	*Pgm*		
24	2	5	2	7	15	20	5	Netherlands (1), United States (1)	1986, 1993
750	2	5	2	9	15	20	5	United Kingdom (2), Germany (1)	1998-1999
181	10	3	15	7	5	41	31	Mali (6), Chad (1), Niger (2), Ghana (1)	1970-2000
182	10	3	15	26	5	41	31	Mali (4)	1970
751	10	3	15	7	8	41	6	Burkina Faso (3), Ghana (12)	1996-2000

### Serologic Results

African isolates of clonal grouping X-I were NT:P1.5. The 11 North American and European isolates of clonal grouping X-II for which serologic data were available were 21:P1.16. Diverse serotype and serosubtype patterns were found for the other isolates from North America and Europe

The PFGE patterns distinguished two finer groups (Ia and Ib) in clonal grouping X-I, which differ consistently in four *NheI* and three *SpeI* fragments (Figure 1). All 14 group Ia strains tested were either ST181 or ST182, which differ at one of the seven gene fragments (Table). All 15 group Ib strains tested were ST751, which differs from ST181 and ST182 at two to three loci (Table). Group Ia included 10 isolates from Mali (1970-1990), 4 isolates from Niger (1997-1998), and the sole isolate from Chad (1995), as well as one of 79 isolates from Ghana (2000). All five isolates from Burkina Faso (1996-1998) and 78 of 79 isolates from Ghana (1998-2000) were in group Ib.

## Discussion

The general population structure of *N. meningitidis* is panmictic as a result of the frequent import of alleles from unrelated Neisseriae (*20*,*23*). Furthermore, several MLST studies have demonstrated that meningococci from healthy carriers are highly diverse (*16*,*26*). Phylogenetic trees of different housekeeping genes from *N. meningitidis* are no more congruent with each other than with random trees (*27*). Our results for sequence typing of housekeeping genes of serogroup X meningococci also fit this pattern. Phylogenetic analysis of allele differences resulted in a bushlike tree that does not seem to contain any deep phylogenetic information. However, two clonal groupings were found in this otherwise panmictic group of bacteria. The same isolates were assigned to both clonal groupings by two independent methods, MLST and PFGE, indicating that these assignments reflect real genetic relationships and do not depend on the methods used.

Similar concordant genetic relationships were discerned in epidemic serogroup A *N. meningitidis* by multilocus enzyme electrophoresis (MLEE), random amplification of polymorphic DNA (RAPD), and MLST; population genetic analyses confirmed that the population structure of these bacteria is clonal (*28*). Concordant groupings were also discerned by MLEE and MLST among the so-called hypervirulent serogroup B and C isolates of the ET-5 complex, ET-37 complex, lineage III, and cluster A4 (*16*). Although their apparent clonality may reflect an epidemic population structure (*23*), this possibility has been excluded for epidemic serogroup A meningococci (*28*). Therefore, multiple clonal groupings exist in *N. meningitidis,* even though the population structure of most of the species is panmictic.

The population structure of subgroup III serogroup A meningococci seems to represent continual, sequential replacement of fit genotypes by related variants during periods of several years to decades (*19*). In subgroup III, nine genoclouds, each consisting of a frequent genotype plus its rarer, less fit variants, have been identified during 3 decades of pandemic spread. Our PFGE data for clonal grouping I of serogroup X suggest that clonal grouping X-I also has a genocloud structure. Two sets of PFGE variants (group Ia and Ib), which might each represent a genocloud, were detected in different countries (Mali, Chad, and Niger; and Burkina Faso and Ghana, respectively). Additional analyses of polymorphic genes are necessary to clarify the uniformity of these groups and to test the similarity of their population structure compared with that of subgroup III.

Both serogroup X clonal groupings described here were isolated over decades, on multiple occasions, and from diverse locations. Clonal grouping X-I (1970-2000) was isolated from different countries in West Africa, and clonal grouping X-II (1986-1999) was isolated from Europe and North America. For clonal grouping X-I in Ghana, the disease rate in healthy carriers was estimated to be 3/10,000 (12). Clonal grouping X-1 is thus of considerably lower virulence than serogroup B ET-5 complex bacteria (disease/carrier rate of 2,100/10,000 [*29*]) or serogroup A subgroup III bacteria during a postepidemic period in a vaccinated population (100/10,000 [*9*]).

The relationship between bacterial fitness and clonality has not yet been investigated extensively in natural isolates. Variation in virulence between bacterial genotypes leads to more uniformity in disease isolates than in carriage organism in *Streptococcus pneumoniae* (*30*) and *Staphylococcus aureus* (*31*). However, our data suggest that the clonal structure of certain meningococcal genotypes need not reflect virulence but rather is associated with genotypes that are particularly fit at colonizing the nasopharynx and spreading from person to person.

Although clonal grouping X-I bacteria are less virulent than serogroup A and B meningococci, they are still pathogenic. Most strains described here were isolated from asymptomatic carriers or patients with rare endemic cases. However, group Ia caused a meningitis outbreak with >60 cases in 1997 in Niger (*11*). Group Ib caused a smaller outbreak in 2000 in Ghana (*12*). These results suggest that X-I meningococci may even be capable of causing epidemics. Meningococci are naturally transformable, and horizontal DNA transfer is frequent in these bacteria (*20*–*22*). Meningococcal carriage is usually low in interepidemic periods in Africa (*1*,*12*,*32*,*33*), offering less opportunity for horizontal genetic exchange, which could account for the low genetic variability in serogroup X meningococci in Africa.

For more than a decade, many countries in the African meningitis belt have vaccinated extensively with A/C polysaccharide vaccines (*34*). Recently, mass vaccination with conjugated serogroup C vaccines has been implemented in the United Kingdom, and strong initial protection has been reported (*35*)**.** However, if effective, these vaccines may well select for the spread of bacteria for which they are not protective (*36*), including unusual causes of disease such as serogroups Y, W135, and X. Capsule switching due to DNA transformation has been documented (*37*,*38*), and effective vaccination against serogroups A and C may select for capsule switch variants of fit genotypes expressing a capsular polysaccharide not included in the vaccination program. The recent outbreaks after the 2000 Hajj pilgrimage, caused by W135 ET-37 complex meningococci (*39*,*40*), may reflect exactly such selection. These findings support the development of comprehensive conjugate vaccines that include capsular polysaccharides from formerly rare causes of disease such as serogroup X.
